# Dilated Cardiomyopathy as a Result of Coxsackie B Virus Myocarditis

**DOI:** 10.7759/cureus.35895

**Published:** 2023-03-08

**Authors:** Inderpal Singh, Sajithaa Varadarasa, Jordan Swisher, Harish Gidda, Theodore Schreiber

**Affiliations:** 1 Department of Internal Medicine, Ascension St. John Hospital, Detroit, USA; 2 Department of Cardiology, Ascension St. John Hospital, Detroit, USA

**Keywords:** dilated cardiomyopathy (dcm), cardiogenic shock, nonischemic cardiomyopathy, coxsackie b virus, viral-induced myocarditis

## Abstract

Dilated cardiomyopathy (DCM) is a myocardial disease that is characterized by left ventricular or biventricular dilation and impairment of systolic function. The etiology is often unknown although it has been thought that DCM may be a consequence of viral myocarditis. The most commonly implicated viruses in the development of myocarditis include coxsackie B virus, hepatitis, parvovirus, cytomegalovirus, influenza virus, and adenovirus. DCM carries a poor prognosis and high rates of mortality, therefore early diagnosis and treatment are imperative.

A 47-year-old male presented with atypical chest pain, along with progressive dyspnea. The patient also endorsed symptoms consistent with acute viral syndrome roughly one week prior to presenting to the hospital. The patient initially presented in cardiogenic shock. An initial workup including an echocardiogram was done and showed an ejection fraction of 10-15% with severe left ventricular and left atrial dilation. Left-sided cardiac catheterization revealed nonobstructive coronary artery disease. The patient was placed on mechanical circulatory and inotropic support and was transferred to the cardiovascular intensive care unit. Cardiac MRI was done and showed a moderately sized pericardial effusion along with signs indicative of myocarditis. Serologic testing was positive for coxsackie B virus type IV antibodies. The patient's clinical picture improved as circulatory and inotropic support was removed and the patient was discharged with close outpatient follow-up and evaluation for cardiac transplant.

## Introduction

Dilated cardiomyopathy (DCM) is a myocardial disease that is characterized by left ventricular or biventricular dilation and impairment of systolic function. The etiology is often unknown although it has been thought that DCM may be a consequence of viral myocarditis due to either direct cardiac cytotoxicity or an adverse autoimmune reaction to remaining viral genomic fragments in the cardiac myocytes [1]. Diagnosis is generally made by exclusion, with findings of cardiomyopathy in the absence of coronary artery disease, valvular or pericardial disorders, and specific heart diseases [2]. Here, we present a case of DCM due to coxsackie B virus myocarditis in a young, otherwise healthy patient.

## Case presentation

A 47-year-old male with a past medical history of attention deficit hyperactivity disorder (ADHD) presented with atypical chest pain, progressive dyspnea, subjective fevers, and arthralgias for one week. On arrival, initial vital signs were significant for sinus tachycardia with a rate of 118 beats/minute, hypotension with a mean arterial pressure (MAP) of 42 mmHg, and hypoxia with an oxygen saturation of 94% on four liters of oxygen given via nasal cannula. An elevated central venous pressure (CVP) of 26 mmHg was also noted. On physical exam, the patient was awake and alert, bilateral pitting peripheral edema was noted in the lower extremities bilaterally and rales were heard in all lung fields upon auscultation. The patient initially presented with signs and symptoms of cardiogenic shock. An initial workup including a transthoracic echocardiogram was done and showed an ejection fraction of 10-15% with severe left ventricular and left atrial dilation and global hypokinesis of the myocardium. Pulmonary artery systolic pressure was also seen to be elevated with a reading of 50 mmHg. Left and right-sided cardiac catheterization was done and revealed nonobstructive coronary artery disease and a decreased cardiac index of 2 L/min/m^2^. The patient was placed on mechanical circulatory support with an Impella device along with inotropic support, with intravenous Milrinone and Dobutamine, and was transferred to the cardiovascular intensive care unit. The initial laboratory evaluation is summarized in Table 1.

**Table 1:** Initial laboratory workup on admission

Test	Results	Reference Range
White blood cell count	14.58	5.00-11.00 x 10^3^/uL
Creatinine	3.50	0.5-1.1 mg/dL
Blood Urea Nitrogen	40	6-23 mg/dL
Bilirubin direct	0.6	0.0-0.8 mg/dL
Bilirubin total	1.6	0.1-1.2 mg/dL
Aspartate aminotransferase	954	1-35 U/L
Alanine aminotransferase	284	1-45 U/L
Brain natriuretic peptide	5012	101 pg/mL
Troponin-T	0.25	<0.05 ng/mL

**Figure 1:**
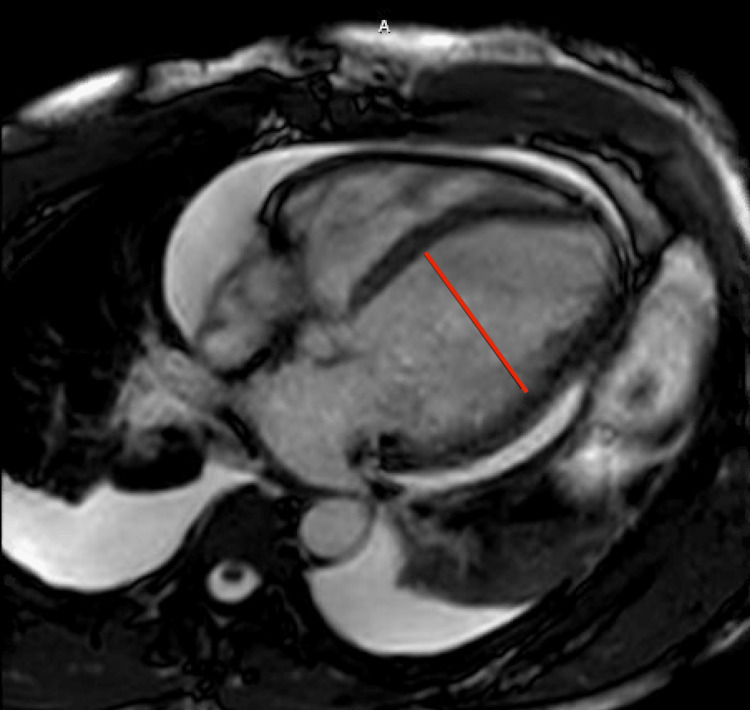
A cardiac magnetic resonance imaging four chamber view showing a dilated left ventricle with an end-diastolic diameter of 6.8 cm as seen with the red line across the short axis of the heart.

**Figure 2:**
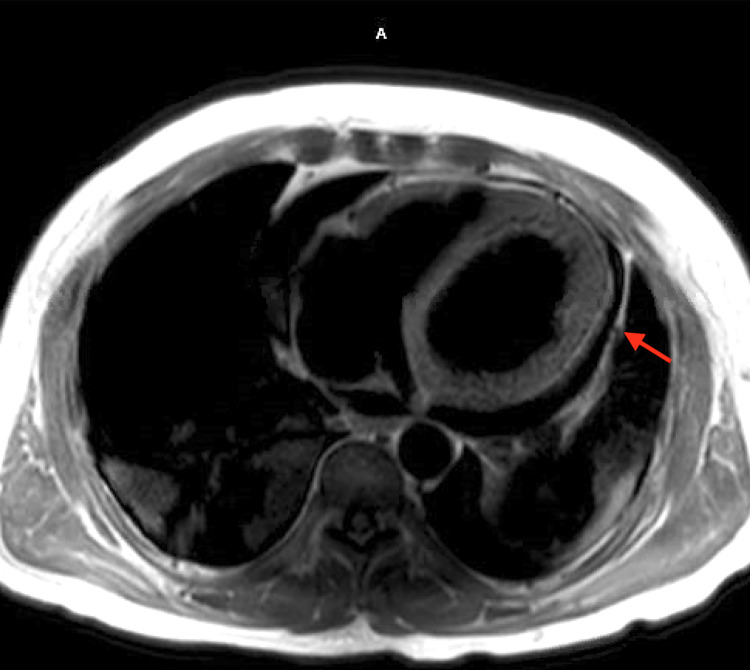
A cardiac magnetic resonance imaging short axis view showing a moderately sized pericardial effusion with slight myocardial enhancement in the inferior lateral wall (arrow).

The patient was initiated on continuous renal replacement therapy (CRRT) due to the rising serum creatinine and decreased urine output of less than 100 mL/hour. A cardiac magnetic resonance imaging (MRI) test was done and showed findings suggestive of severe systolic dysfunction with a left ventricular end-diastolic diameter of 6.8 cm (Figure 1) along with findings significant of myocarditis with a mild to moderate pericardial effusion (Figure 2). As outlined in Table 2, further work up to elucidate the etiology for cardiomyopathy, including C3 and C4 complement levels, and serologies of human immunodeficiency virus, hepatitis, cytomegalovirus, influenza, parvovirus, COVID-19, and Lyme disease were negative. Of note, serologies of coxsackie B virus type IV were positive with titers of 1:160.

**Table 2:** Infectious and extra-cardiac workup to elucidate cause of cardiomyopathy Ab - Antibody, IgG - Immunoglobulin G, IgM - Immunoglobulin M, PCR - Polymerase chain reaction, HIV - Human immunodeficiency virus

Test	Results	Reference Range
C3 Complement	188	79-152 mg/dL
C4 Complement	53	16-38 mg/dL
Hepatitis A, B core and C total Ab	Negative	Negative
Coxsackie B virus type IV Ab	1:160	<1:10
Lyme disease IgG, IgM Ab	Negative	Negative
Parvovirus B19 IgG Ab	0.05	<0.90 IV
Parvovirus B19 IgM Ab	0.10	<0.90 IV
Adenovirus nucleic acid on PCR	Not detected	Not detected
Influenza A and B nucleic acid on PCR	Not Detected	Not detected
Cytomegalovirus IgG, IgM	0.2	0-0.8 AI
HIV-1, HIV-2 Ab	Nonreactive	Nonreactive

Over the course of the patient’s admission, the patient’s clinical picture improved. The patient was weaned off of mechanical circulatory and inotropic support. The patient was also transitioned from CRRT to conventional hemodialysis. The patient was then initiated on guideline-directed medical therapy (GDMT) with metoprolol succinate, hydralazine, and isosorbide dinitrate. The patient was then transferred to an outside facility for higher-level care, along with further workup and evaluation for cardiac transplantation. Upon contacting the patient, the patient has since received a left ventricular assist device as a bridge for a tentative plan to undergo cardiac transplantation at a later time.

## Discussion

DCM is a primary cardiac disease of unknown cause characterized by left ventricular or biventricular dilation and impaired myocardial contractility [3]. The usual clinical presentation of DCM includes symptoms and features of heart failure, arrhythmias, or thromboembolism. Numerous etiologies have been identified, with viral infection being the most common, causing preceding myocarditis that can lead to the development of DCM. The most commonly implicated viruses in the development of myocarditis include the Coxsackie B virus, hepatitis, parvovirus, cytomegalovirus, influenza virus, and adenovirus among others [4]. This was seen in our patient with the initial symptoms consistent with acute viral illness roughly one week prior to presenting to the hospital and the subsequent increase in coxsackie B virus type IV antibody titers.

Diagnosis is generally made by exclusion in the absence of coronary artery disease, valvular or pericardial disorders, and specific heart diseases [2]. Initial diagnostic evaluation should include a thorough history and physical exam, along with laboratory testing including an electrocardiogram, and serum troponin levels. Cardiac imaging should also be done including an echocardiogram and cardiac MRI, which can aid in establishing a diagnosis when no clear cause for cardiomyopathy is evident. In select cases, patients with suspected myocarditis who exhibit signs of hemodynamic compromise, left or right-sided cardiac catheterization can also be done to aid in the diagnostic workup. Definitive diagnosis is made by endomyocardial biopsy when other causes of cardiomyopathy have been excluded [5]. Despite this, many patients may not be suitable candidates for endomyocardial biopsy, as was the case for this patient due to being clinically unstable. As such, a diagnosis of clinically suspected myocarditis can be made on the basis of clinical presentation in the absence of other etiologies of cardiovascular disease, such as coronary artery disease, valvular disease, and extra-cardiac causes.

The pathogenesis and mechanism by which viral myocarditis leads to DCM are suspected to be due to either direct cardiac cytotoxicity via entry of the virus into cardiac myocytes or by remaining genomic fragments in the cardiac myocytes leading to an adverse autoimmune response [2]. This is supported in the literature as infectious viruses can no longer be detected in the myocardium by endomyocardial biopsy after approximately 14 days post-infection, although genomic fragments, such as ribonucleic acid (RNA) of the virus, are still detectable in the myocardium passed this time [6-7]. In spite of these findings, pathological examination through biopsy regularly shows evidence of inflammation and patterns consistent with the activation of immune cells such as macrophages, T-helper cells, and antibody-forming B cells [8]. Through the formation and deposition of immune complexes of autoantibodies with complement factors and viral components, along with the release of cytokines, underlying collagen deposition with cardiac remodeling and fibrosis occurs. This response to the underlying inflammatory process leads to the findings typically seen in DCM, such as regional or global myocardial dysfunction and left or biventricular ventricular dilation [9].

Treatment, once DCM has developed, is limited and is generally directed toward symptom management. Prognosis is poor as, in the literature, 40 to 50% of patients who develop DCM die within two years of diagnosis, with the main cause of death being due to cardiogenic shock or fatal cardiac arrhythmias [4]. Conventional treatment with diuretics and GDMT for congestive heart failure with reduced ejection fraction has no effect on the progression of the disease and has not been shown to improve mortality in patients with DCM [4]. Treatment with antiarrhythmics also has not been shown to improve mortality or survival except in patients who present with episodes of sustained ventricular tachycardia or predominantly right ventricular dilation [10]. The only treatment option that has been shown to improve prognosis, and mortality is cardiac transplantation, and, as such, early diagnosis and evaluation for cardiac transplantation is critical [11].

## Conclusions

DCM is a nonischemic cardiomyopathy that carries a poor prognosis and high mortality rates. Viral infections are a common cause of DCM due to the development of preceding myocarditis, with the most commonly implicated viruses being coxsackie B virus, hepatitis, parvovirus, cytomegalovirus, influenza, and adenovirus. In otherwise healthy patients with nonischemic cardiomyopathy, viral etiologies must be suspected and investigated as prompt diagnosis and treatment with implantable left ventricular assist devices and subsequent cardiac transplantation can improve mortality.
